# Omphalocele and Gastroschisis in Newborns: Over 16 Years of Experience from a Single Clinic

**DOI:** 10.21699/jns.v6i2.530

**Published:** 2017-04-15

**Authors:** Shunusuke Watanabe, Tatuya Suzuki, Fujio Hara, Toshihiro Yasui, Naoko Uga, Atuki Naoe

**Affiliations:** Department of Pediatric Surgery, Fujita Health University, Japan

**Keywords:** Omphalocele, Abdominal wall tear, Assisted reproductive technology, Closed gastroschisis

## Abstract

Infants born with potentially life-threatening conditions of omphalocele and gastroschisis may require long-term hospitalization. We aimed to compare the outcomes of these two conditions occurring over a 16-year period (2001-16). It is a retrospective study of 19 newborns undergoing surgery for these two abdominal wall defects (8 patients with omphalocele and 11 cases of gastroschisis). The average birth weights for the newborns with omphaloceles and gastroschisis were 2554.5 g and 2248.6 g respectively. Associated anomalies included trisomy 18, Beckwith-Wiedemann syndrome, congenital heart disease, Meckel’s diverticulum, inguinal hernias, renal deformities, limb deformities, cryptorchidism, body stalk anomalies, and closed gastroschisis. The average hospital stay for the newborns with omphaloceles and gastroschisis were 42.6 days 50.2 days respectively. The time to the start of postoperative nutritional supplementation for the newborns with omphaloceles and gastroschisis were 4.3 days for the infants with omphaloceles and 7.3 days for respectively. The survival rates for the newborns with omphaloceles and gastroschisis were similar, 87.5% and 81.8% respectively. Survival rates in omphalocele correlated negatively with associated anomalies. In gastroschisis cases, strict care is necessary when intestinal dilation is observed via fetal sonography.

## INTRODUCTION

Of the various types of abdominal wall dysplasia, omphalocele and gastroschisis are relatively frequent among newborn surgical conditions. In recent years, we have seen the development of preoperative diagnosis

including maternal serum screening and fetal ultrasound [1-3]. Omphalocele is caused by failure of the four mesodermal-derived folds (left, right, cranial and caudal) to fuse at approximately 3 to 4 weeks of embryonic development (abdominal wall formation failure theory). Gastroschisis occurs when the expanding intestinal tract becomes unable to secure a space inside the umbilical corpus cava owing to the disrupted development of the umbilical cavity. Absorption of the right umbilical vein is thought to weaken the body wall to the right of the umbilicus. The prevalence of omphalocele is reported to be 0.6–2.2 per 10,000 births [4-6], whereas the prevalence of gastroschisis is reported to be 0.5–4.4 per 10,000 births [5-8]. Newborns with abdominal wall defects frequently endure prolonged hospitalizations, with resulting complications in nutritional condition and incidence of infections.

We report the results of a comparative series of omphalocele and gastroschisis cases that were admitted to the neonatal intensive care unit (NICU) at our hospital during the 16-year period from 2001 to 2016.

## Materials and Methods

This retrospective study included 8 neonates with omphalocele and 11 neonates with gastroschisis surgeries performed over 16 years (2001 to 2016) in the Department of Pediatric Surgery at our hospital. We evaluated these patients in terms of demographics, prenatal diagnosis, preoperative status, associated complicating deformities, surgery performed, length of hospitalization, postoperative enteral nutritional supplementation time, postoperative complications, and mortality.

We utilized the likelihood ratio test for statistical analysis, and we used JMP 12.2 (SAS Institute Inc, Cary, NC, USA). A *p*-value of <0.05 was considered statistically significant. 

## RESULTS

**Figure 1:**
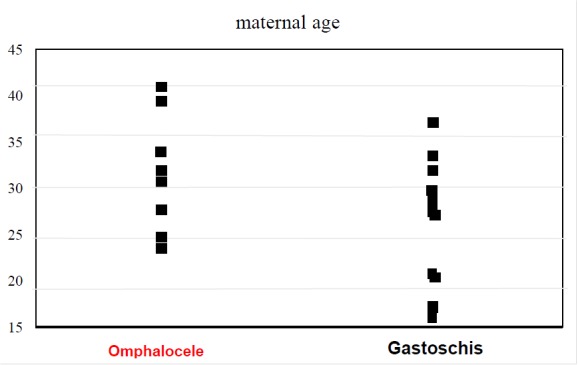
Maternal age in omphalocele and gastroschisis.

The average birth weight among the neonates with omphalocele was 2554.5 g (1692 g to 4420 g). There were five cases of infants with low birth weight (1500 g to 2499 g). The average 5-minute Apgar score was 8.8. The average gestation period was 37.4 weeks. There were 7 cases of premature birth (31 weeks to 39 weeks), and one case of mature birth. The average maternal age was 31.6 years (24-40 years). ART was involved in 1 case. Two cases involved natural births, and six cases were delivered by cesarean section. The ratio of males to females was 1:3 (two males) (Table 1, Fig. 1).

The average birth weight of the infants with gastroschisis was 2248.6 g (1475 g to 3000 g), including six cases of low birth weight (1500 g to 2499 g) and one case of extremely immature infants (1000 g to 1499g). The average 5-minute Apgar score was 7.5. The average gestation period in weeks was 36.5 weeks. All neonates were born prematurely (36 weeks to 37 weeks). The average maternal age was 25.6 years (17–37 years old). ART was not involved. Two cases were natural births, and nine cases were delivered via cesarean section. The ratio of males to females was 2.7:1 (Table 1, Fig. 1). 

For statistical assessment, the likelihood ratio test was performed for infants with either omphalocele or gastroschisis: Birth weight (p < 0.01); 5-minute Apgar score (p = 0.73); gestation (p = 0.55) maternal age (p = 0.98); 5-minute Apgar score (p = 0.73); gestation period (p = 0.55), maternal age (p = 0.03); cesarean section (p = 0.98) (Table 1, Fig. 1).

Antenatal diagnosis: Prenatal diagnosis was made in 17 of the 19 cases. One patient with omphalocele and 1 patient with gastroschisis were not diagnosed prenatally.

Preoperative conditions: Four cases of herniated liver were discovered among the omphalocele cases. Four additional cases involved isolated herniation of the intestinal tract. Prophylactic antibiotics were administered prior to surgery in all patients who did not exhibit cervical closure immediately after birth. Pressors were administered in two cases. Among the gastroschisis cases, herniated organs other than those in the intestinal tract included one herniated bladder and one herniated liver. None of these newborns received pressors prior to surgery.

**Figure 2:**
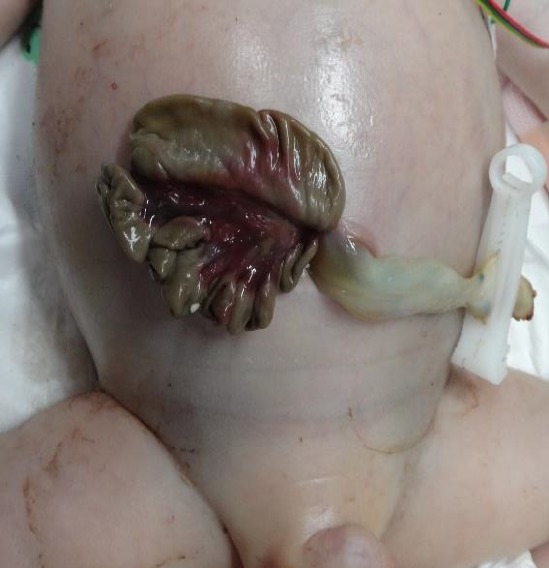
Closing gastroschisis.

Associated anomalies: The neonates with omphalocele were associated with the following anomalies: Beckwith–Wiedemann syndrome (n=2), congenital heart disease (n=2), Meckel’s diverticulum (n=2), trisomy 18 (n=1), inguinal hernia (n=1), renal deformity (n=1), and limb deformity (n=1). The neonates with gastroschisis were associated with the following anomalies: cryptorchidism (n=1), body stalk anomaly (n=1), and closed gastroschisis (n=1) (Table 2). The later had a 7 mm wide deformed abdominal opening, in which the entire herniated intestine was necrotic (Fig. 2). 

Surgery: Primary closure was achieved among 7/8 neonates with omphalocele: one neonate required secondary closure. Primary closure was achieved among 6/11 neonates with gastroschisis. Three neonates required secondary closure and two cases could not be closed. All defects that could not be closed primarily were addressed using the Allen-Wrenn (silo) method [9].

Postoperative conditions and outcomes: The length of hospitalization was 42.6 days in the omphalocele series and 50.2 days in the gastroschisis series. Postoperative enteral nutritional supplementation was initiated at 4.3 days (2 to 8 days) in the omphalocele series, and 7.3 days (3 to 16 days) in the gastroschisis series. Postoperative enteral nutritional supplementation within five days was shorter in the length of hospitalization with the gastroschisis series (p<0.05). Postoperative complications in the omphalocele series included one case of heart failure, one case of chronic lung disease, and one case of cerebral hemorrhage. In the gastroschisis series, postoperative complications included one case of pneumonia, one case of pleural effusion, one case of heart failure, one case of chronic lung disease, one case of cerebral hemorrhage, and one case of septicemia. The survival rate in the omphalocele series was 87.5%, with one patient death. This death occurred in a neonate of trisomy 18 with complex cardiac deformity. The survival rate in the gastroschisis series was 81.8%, with two patient deaths; one neonate with a body stalk anomaly had postoperative sepsis, while the other death occurred as a result of closed gastroschisis. In that case, the intestinal tract was already necrotic and inoperable.

## DISCUSSION 

There was no significant difference between the omphalocele and gastroschisis series with regard to 5-minute Apgar score, or gestational age. However, birth weight was significant higher for the omphalocele neonates as compared those with gastroschisis. Low birth weight in infants with gastroschisis has been known as a factor associated with perinatal mortality [10]. 

A significant difference was observed between the omphalocele and gastroschisis series with respect to maternal age; mothers in the gastroschisis series tended to be younger. In another published series, many gastroschisis cases involved mothers aged less than 25 years (and especially with mothers aged less than 20 years) [5]. 

Although the issue of optimal method of delivery for neonates in omphalocele and gastroschisis is a subject of debate, elective cesarean section should be avoided, as there is no difference in mortality rate related to delivery method [11,12]. All neonates delivered vaginally in our study survived, probably because they didn’t have life-threatening associated anomalies. 

Our series included one infant with closed gastroschisis. Closed gastroschisis has been reported to occur in 6% of cases [13]. This report also states that defective openings in the abdominal wall can shrink or close during the fetal period, leading to ileus and gastrointestinal necrosis, resulting in a very high mortality rate [13]. Treatment methods include resection of the necrotized intestinal tract during the closure procedure. As a result, short bowel syndrome may occur, and it has been reported that small intestinal transplantation may be required in such cases [14]. Similar to our experience, prenatally diagnosed gastroschisis cases have been known to intestinal dilatation earlier too [15, 16]. The possibility of closed gastroschisis should be taken into account in these cases.

Total hospital stays, length of stays in NICU were longer and postoperative enteral nutritional supplementation was administered earlier in the neonates with gastroschisis as compared with the neonates with omphalocele. Earlier start of postoperative enteral nutritional supplementation has been known to lead to faster recovery in gastroschisis patients [15]. 

Omphalocele is known to be associated with poorer prognosis when accompanied by associated anomalies, especially in presence of genetic abnormalities [4]. We had similar observations. 

Our study has several limitations. The study involved patients in only one facility. The neonates that did not reach the NICU could not be investigated.

## CONCLUSIONS

In addition to the increased incidence of gastroschisis in comparison to omphaloceles, initiation of postoperative nutritional supplementation was later in the gastroschisis series and hospitalization periods tended to be longer. 

Regarding prognosis, the outcomes for infants with omphaloceles depended on the presence of complex associated anomalies. The neonates with gastroschisis had relatively favorable prognosis provided the defect could be closed. Initiation of postoperative enteral nutritional supplementation at an early stage may be desirable. Stricter surveillance is necessary in gastroschisis cases when intestinal dilation is observed via fetal ultrasound.

## Footnotes

**Source of Support:** None

**Conflict of Interest:** None

## Figures and Tables

**Table 1:** Omphalocele and Gastroschisis (2001–2016)

	Omphalocele(n=8)	Gastroschisis (n=11)	p value
Birth weight(g)	2554.5(1692-4420)	2248.6(1475-3000)	p<0.01
5-MinuteAPGARvalue	8.8(7-10)	7.5(0-9)	p=0.73
Gestation period(w)	37.4(31-40)	36.4(36-38)	p=0.55
Maternalage(y)	31.6(24-40)	25.6(17-37)	p=0.03
Cesarean section	6	9	p=0.98
M:F	2:6	8:3	p<0.05

**Table 2:** Associated anomalies

Congenital anomaly	Omphalocele(n=7/8)
Trisomy 18	1=
Beckwith-Wiedemann syndrome	2
Congenital Heart Disease	2
Meckel's diverticulum	2
Inguinal hernia	1
Congenital renal anomaly	1
Cacomelia	1
**Congenital anomaly**	**Gastroschisis (n=3/11)**
Undescended testicle	1
Bodystalk anomaly	1
Closinggastroschisis	1

## References

[1] Sebire NJ, Spencer K, Noble PL, Hughes K, Nicolaides KH (1997). Maternal serum alpha-fetoprotein in fetal neural tube and abdominal wall defects at 10 to 14 weeks of gestation. Br J Obset Gynaecol.

[2] Grande M, Arigita M, Borobio V, Jimenez JM, Fernandez S, Borrell A (2012). First-trimester detection of structural abnormalities and the role of aneuploidy markers. Ultrasound Obset Gynecol.

[3] Joo JG, Castlos E, Rigo Jr J (2010). Abdominal wall malformations in a 15-year fetopathological study: accuracy of prenatal ultrasonography diagnosis. Prenat Diagn.

[4] Marshall J, Salemi JL, Tanner JP, Ramakrishnan R, Feldkamp ML, Marengo LK  (2015). Prevalence, Correlates, and Outcomes of Omphalocele in the United States, 1995-2005. Obstet Gynecol.

[5] Suita S, Okamatsu T, Yamamoto T, Handa N, Nirasawa Y, Watanabe Y  (2000). Changing profile of abdominal wall defects in Japan: results of a national survey. J Pediatr Surg.

[6] Tan KB, Tan KH, Chew SK (2008). Gastroschisis and omphalocele in Singapore: a ten-year series from 1993 to 2002. Singapore Med J.

[7] Nazer Herrera J, Karachon Essedin L, Cifuentes Ovalle L, Assar CR (2016). Gastroschisis: A pandemic with increasing rates? ECLAMC experience in Chile 1982-
2015. Rev Chil Pediatr.

[8] Kirby RS, Marshall J, Tanner JP, Salemi JL, Feldkamp ML, Marengo L  (2013). Prevalence and correlates of gastroschisis in 15 states, 1995 to 2005. Obstet Gynecol.

[9] Kidd JN Jr, Jackson RJ, Smith SD, Wagner CW (2003). Evolution of staged versus primary closure of gastroschisis. Ann Surg.

[10] Calcagnotto H, Müller AL, Leite JC, Sanseverino MT, Gomes KW, Magalhães JA (2013). Associated factors for perinatal mortality in gastroschisis. Rev Bras Ginecol Obstet.

[11] Abdel-Latif ME, Bolisetty S, Abeywardana S, Lui K (2008). Mode of delivery and neonatal survival of infants with gastroschisis in Australia and New Zealand. J Pediatr Surg.

[12] Lurie S, Sherman D, Bukovsky I (1999). Omphalocele delivery enigma: the best mode of delivery still remains dubious. Eur J Obstet Gynecol Reprod Biol.

[13] Houben C, Davenport M, Ade-Ajayi N, Flack N, Patel S (2009). Closing gastroschisis: diagnosis, management, and outcomes. J Pediatr Surg.

[14] Dennison FA (2016). Closed gastroschisis, vanishing midgut and extreme short bowel syndrome: Case report and review of the literature. Ultrasound.

[15] Davenport M, Haugen S, Greenough A, Nicolaides K (2001). Closed gastroschisis: Antenatal and postnatal features. J Pediatr Surg.

[16] Harris J, Poirier J, Selip D, Pillai S, Shah AN, Jackson CC  (2015). Early Closure of Gastroschisis After Silo Placement Correlates with Earlier Enteral Feeding. J Neonatal Surg.

